# Engineered mesenchymal stem-cell-sheets patches prevents postoperative pancreatic leakage in a rat model

**DOI:** 10.1038/s41598-017-18490-9

**Published:** 2018-01-10

**Authors:** Seong-Ryong Kim, Hye-Jin Yi, Yu Na Lee, Ji Yoon Park, Robert M. Hoffman, Teruo Okano, In Kyong Shim, Song Cheol Kim

**Affiliations:** 1 Department of Surgery, Division of HBP and Liver Transplantation, Korea University Anam Hospital, Seoul, Korea; 2 Asan Institute for Life Sciences, Asan Medical Center, University of Ulsan College of Medicine, Seoul, Korea; 3 Department of Chemistry, Wesleyan University, Connecticut, United States; 4 Department of Surgery, University of California, San Diego, CA USA; 5 AntiCancer Inc., San Diego, CA USA; 6 Institute of Advanced Biomedical Engineering and Science, Tokyo Women’s Medical University, Tokyo, Japan; 7 Department of Surgery, Asan Medical Center, University of Ulsan College of Medicine, Seoul, Korea

## Abstract

Post-operative pancreatic fistula (POPF) following pancreatic resection is a life-threatening surgical complication. Cell sheets were prepared and harvested using temperature-responsive culture dishes and transplanted as patches to seal POPF. Two different mesenchymal stem cell (MSC) sheets were compared in terms of the preventative ability for pancreatic leakage in a rat model. Both rat adipose-derived stem cell (rADSC) and bone marrow-derived stem cell (rBMSC) sheets were transplanted. Those rADSC and rBMSC sheets are created without enzymes and thus maintained their cell-cell junctions and adhesion proteins with intact fibronectin on the basal side, as well as characteristics of MSCs. The rats with post-pancreatectomy rADSC- or rBMSC-sheet patches had significantly decreased abdominal fluid leakage compared with the control group, demonstrated by MR image analysis and measurement of the volume of abdominal fluid. Amylase level was significantly lower in the rats with rADSC-sheet and rBMSC-sheet patches compared with the control groups. The rADSC sheet patches had increased adhesive and immune-cytokine profiles (ICAM-1, L-selectin, TIMP-1), and the rBMSC sheets had reduced immune reactions compared to the control. This is first project looking at the feasibility of tissue engineering therapy using MSC-sheets as tissue patches preventing leakage of abdominal fluid caused by POPF.

## Introduction

A postoperative pancreatic fistula (POPF) following pancreatectomy is characterized by leakage of digestive enzymes from postoperative and/or otherwise damaged pancreas. A POPF can lead to dissolution of surrounding organs and blood vessels, hemorrhage, and sepsis^1^. While mortality rates from pancreatic surgery have been declining due to improvements in surgical intervention and medical technology, pancreatic fistula still occurs at a high rate of 10–40%^2^. There are several techniques for preventing leakage of pancreatic secretions, including handsewn sutures, staples^3^, or surgical adhesive materials^4,5^. Several materials including fibrin glue and polyglycolic acid felt are widely used in clinical settings. However, a definitive approach that prevents pancreatic fistula is still lacking^6,7^. Materials currently in use do not completely prevent pancreatic fistula, as they do not actively induce pancreas regeneration, have limited elasticity, and are difficult to attach to irregularly-shaped organs. Ideal materials for preventing pancreatic fistula should enhance tissue regeneration, wound healing, elasticity, and adhesiveness to the pancreatic resection margin.

**Figure 1:**
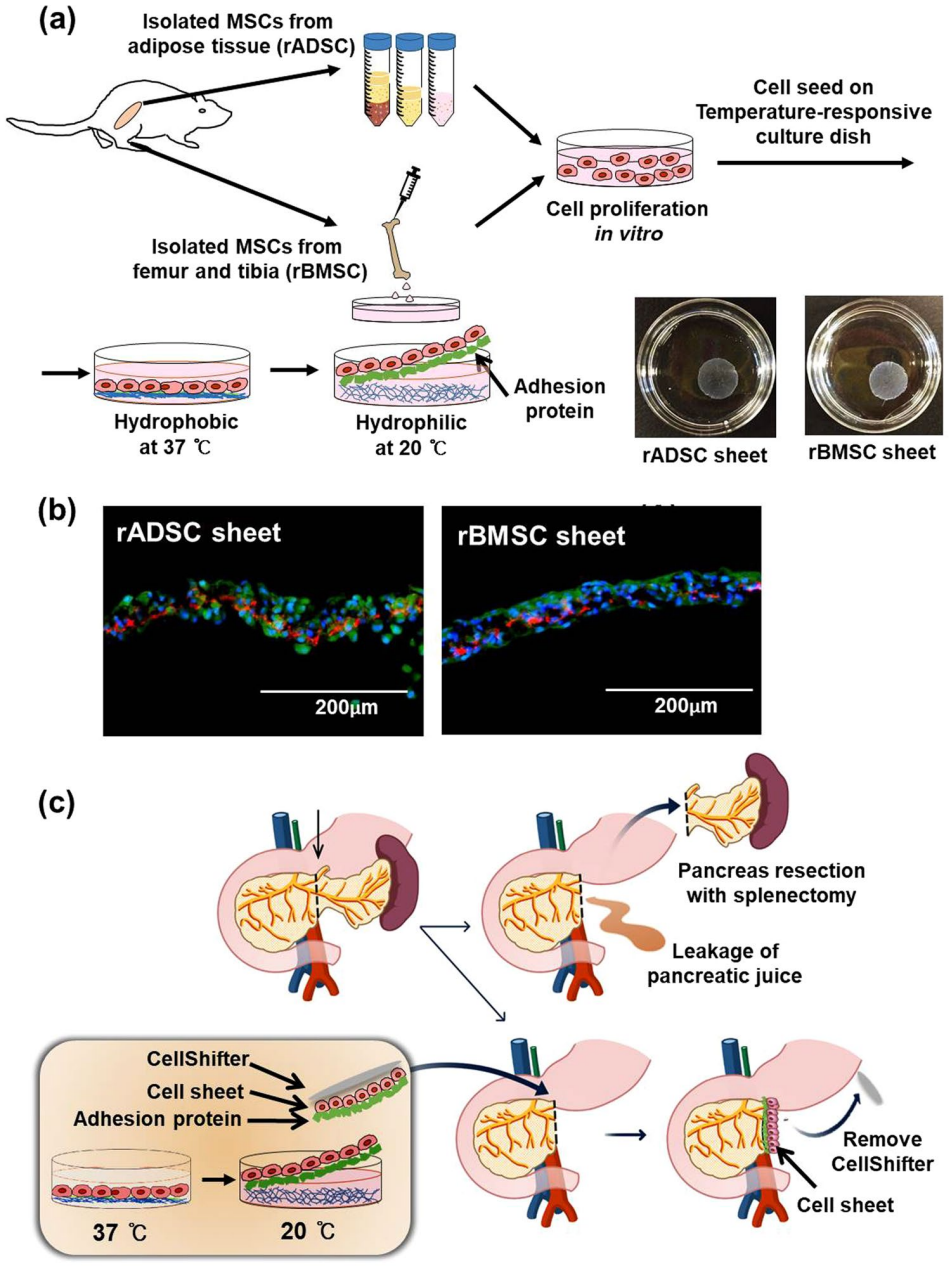
Study design of mesenchymal-stem cell-sheet patches for preventing postoperative pancreatic fistula (POPF). (**a**) Schematic diagram of rADSC and rBMSC isolation from rats, and rADSC and rBMSC sheet formation using temperature-responsive culture dishes. rADSC and rBMSC were cultured for two days and were allowed to reach confluence. At this point, the culture temperature was lowered from 37 °C to 20 °C to harvest the stem-cell sheets. (**b**) Extracellular matrix accumulation by the rADSC and rBMSC sheets. Fibronectin was detected by immunofluorescence. Scale bars represent 200 µm. Green fluorescence: rADSC or rBMSC from GFP-expressing transgenic rats. Blue: nucleus, Red: fibronectin. (**c**) Schematic description of the experimental procedure of the distal pancreatectomy (DP) pancreas resection model and application of stem-cell-sheet patches.

Technologies that use cells to induce wound healing or tissue regeneration are rapidly progressing^8–10^, especially with the use of mesenchymal stem cells (MSC) that have functions in both regeneration and immune response. Recently, several clinical applications of MSC have been reported. While traditional cell therapies often rely on direct single cell injection, this delivery method is impractical for application onto topical regions due to massive cell loss and the low survival rates of single cells *in vivo*. Therefore, new techniques for efficient cell transplantation are needed, and scaffold-free cell sheets are a prevalent cell therapy method used clinically. Conventional cell therapy methods harvest cells using a chemical treatment such as trypsin-EDTA and dispase, or physical force, which leads to cells losing their inherent properties. Cell sheets, however, are harvested simply by changing the culture temperature, thus enabling them to maintain cell-cell connections and adherence protein layers that facilitate attachment to tissue surfaces^11,12^. Successful regeneration of cornea and esophagus using cell sheet technology in clinical trials has been previously reported^13–15^. In the present report, we used mesenchymal stem cell sheets to prevent pancreatic leakage after pancreas surgery in rat models. The process of MSC sheet preparation and application to the pancreas resection site is illustrated in Fig. 1.

## Results

### Formation and characterization of mesenchymal stem cell sheets

**Figure 2:**
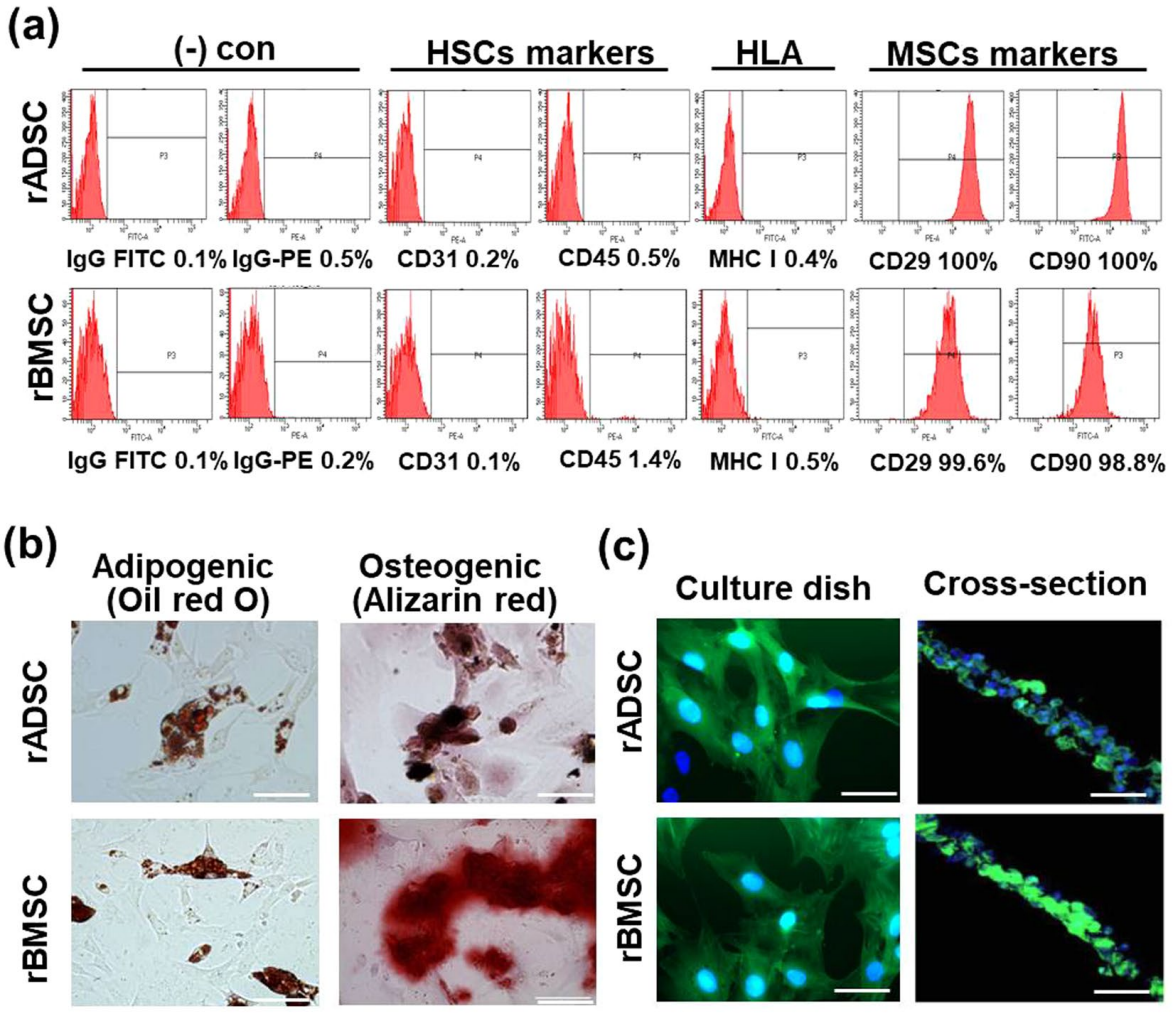
Stem-cell characteristics of rADSC and rBMSC obtained from GFP transgenic rats. (**a**) The expression of surface antigens on rADSC and rBMSC analyzed by flow cytometry. (**b**) Oil-Red O and alizarin-red staining of rADSC and rBMSC. Scale bars represent 200 μm. (**c**) Fluorescence images of mesenchymal stem cells from GFP transgenic rats and the cross-section of a cell sheet. Green: rADSC or rBMSC from GFP transgenic rats, Blue: nucleus.

To confirm whether the cultured rADSCs and rBMSCs maintained mesenchymal-stem cells characteristics, flow cytometry was performed with surface marker antibodies. The results were positive for CD29 and CD90 and negative for CD31, CD45, and MHC class 1 (Fig. 2a). Mesenchymal stem cells (MSC) derived from rat adipose tissue and bone marrow is able to undergo adipogenesis and osteogenesis (Fig. 2b). Adipogenesis and osteogenesis were verified with oil red O staining and alizarin red staining, respectively, in the stem cell sheets. The lipid droplets formed in the adipogenically-induced rat mesenchymal stem cells were stained red with oil red O and calcium deposits were stained red by alizarin red in the osteogenically-induced cells, thereby confirming adipogenesis and osteogenesis of the cultured rat MSC sheets. In order to track the sheets at the transplantation site, we used rADSC and rBMSC from GFP transgenic rats (Tg-CAG-GFP). Strong green fluorescence was observed in the cells and sheets formed from rADSC and rBMSC (Fig. 2c).

### Establishing rat model of pancreatic leakage after pancreatic surgery

**Figure 3:**
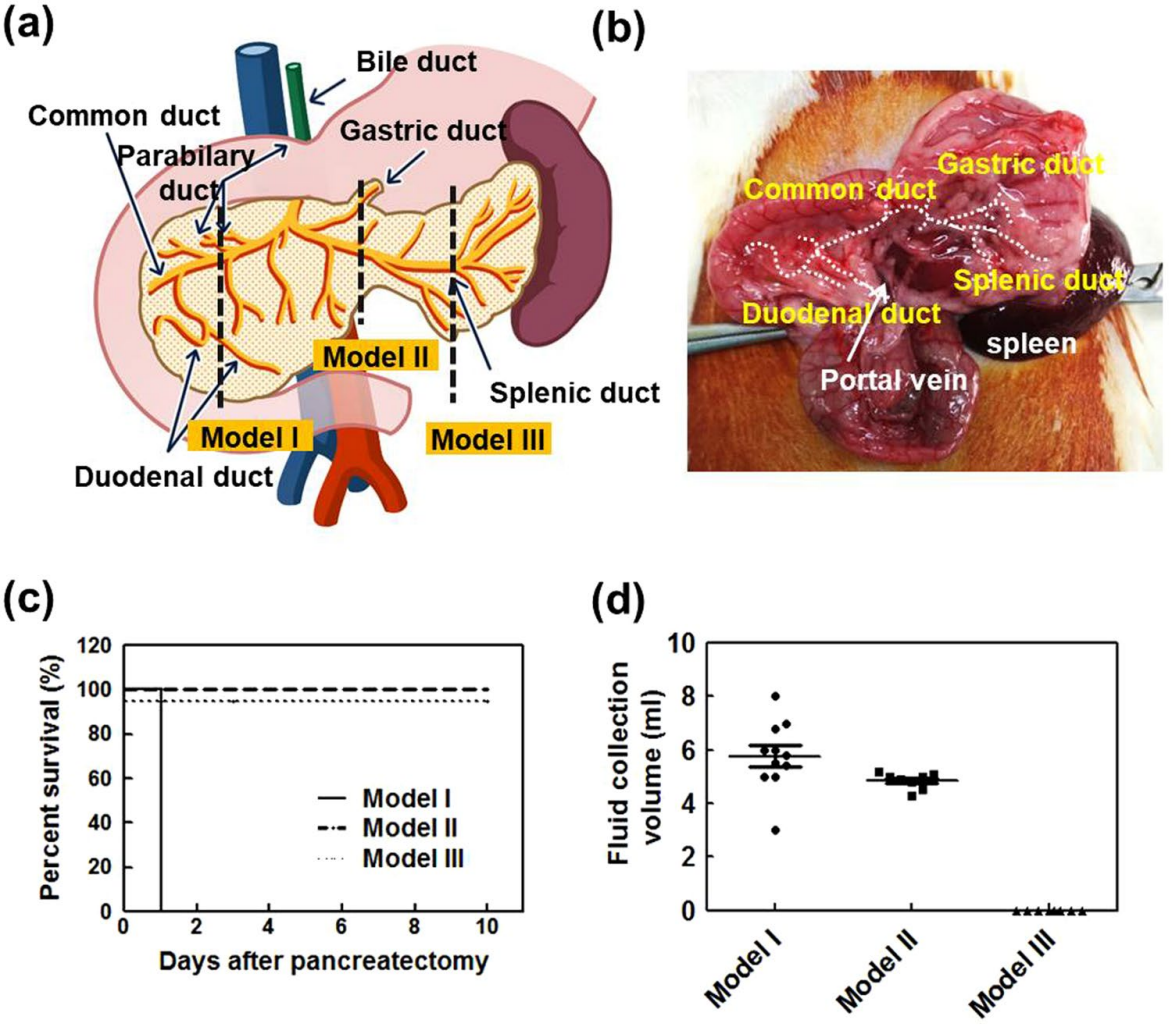
Establishment of a rat model of postoperative pancreatic fistula (POPF). (**a**) Schematic representation of rat pancreas and the resection sites of models I, II, and III. Model I (common pancreatic duct division): division of the pancreas where the common duct of the pancreas and bile duct intersect, Model II (gastric and splenic duct division with distal pancreatectomy and splenectomy): division of both gastric duct and splenic ducts at the left margin of the portal vein. Model III (splenic duct division with distal pancreatectomy and splenectomy): removal of the splenic duct at the pancreas-tail level. (**b**) Photos of the rat pancreas, pancreatic duct, and portal vein. (**c**) Survival rates of models I, II, and III (n = 10 each). While all model I rats died on day 1 after pancreatectomy, all model II and III rats survived during the experimental period. (**d**) The abdominal fluid volume of each model on day 1 after pancreatectomy (n = 10 each).

Figure 3a and b show the resection sites in the rat pancreas. Three different models were developed: model I (common pancreatic duct division), model II (gastric and splenic duct division with distal pancreatectomy and splenectomy), and model III (splenic duct division with distal pancreatectomy and splenectomy). Each model’s survival rate and accumulated abdominal fluid volume at 10 days after distal pancreatectomy (DP) were analyzed and are shown in Fig. 3c and d, respectively. In model I rats, 5.77 ± 1.29 fluid was observed due to post-operative pancreatic fistula (POPF) and all rats died on day 1 (n = 10). Model III rats survived for the whole experimental period (10 days), and no fluid was observed during or after surgery. Model II rats survived for the whole experimental period, with continuous secretion of pancreatic juice, with 4.85 ± 0.31 ml fluid observed on day 1 (n = 10). Thus, model II—hereafter referred to as distal pancreatectomy (DP)—was deemed as the adequate model for assessing the efficacy of materials for prevention of pancreatic leakage.

### Attachment of cell sheets on the pancreas resection surface

**Figure 4:**
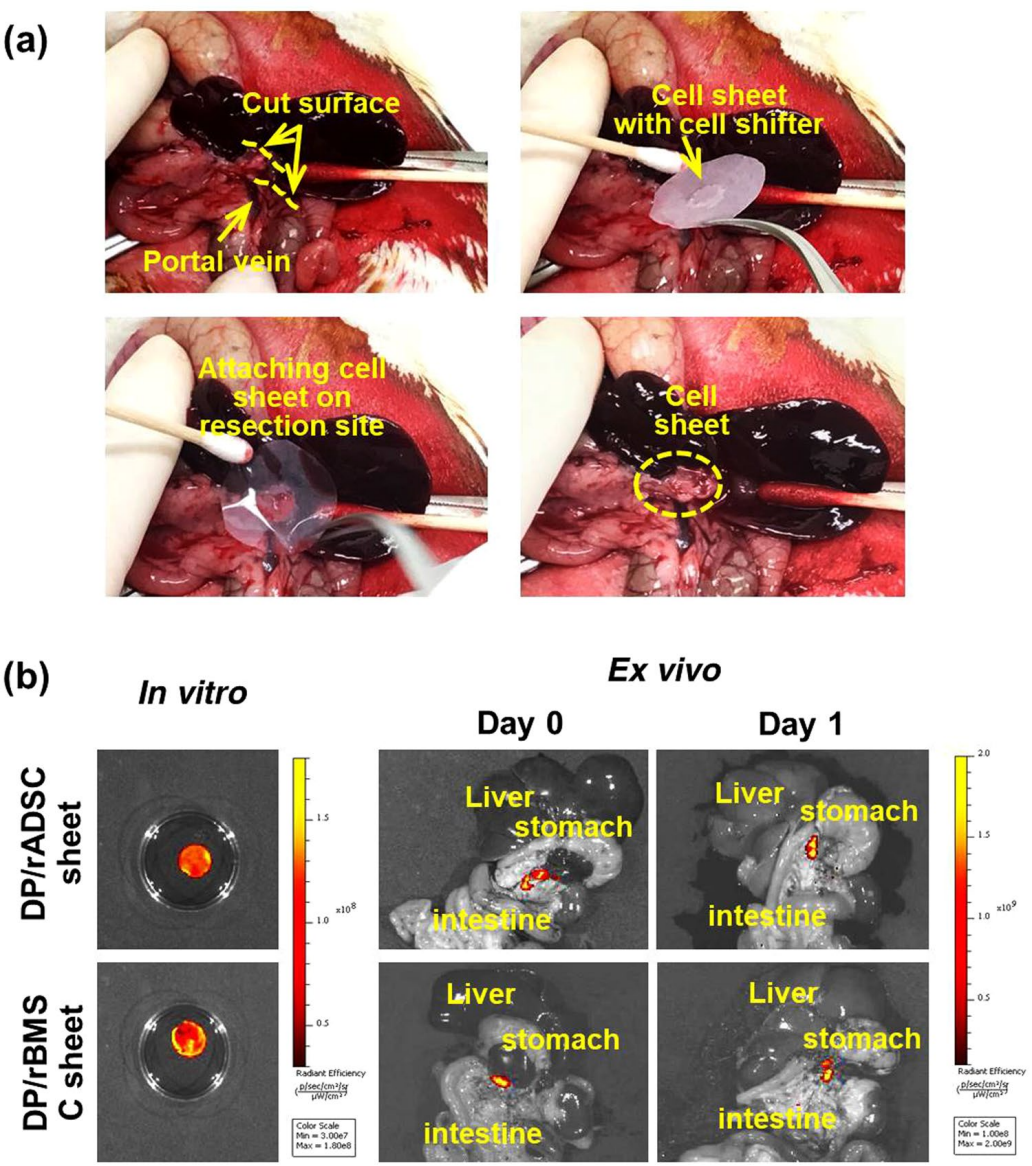
Attachment of stem-cell-sheet patch onto the pancreas resection surface. (**a**) Photos of the DP procedure and application of stem cell-sheet patches onto the resection site. Pancreatic juice was secreted only where the duct was opened (upper left). Preparation of stem cell-sheet patches using CellShifter^TM^ (upper right). Following pancreas resection, the stem-cell patch was attached on the pancreatic resection surface (lower left). After five minutes, the shifter was removed, and only the cell sheet remained (lower right). (**b**) Representative image of GFP fluorescence of a cell sheet *in vitro* and *ex vivo*. rADSC- and rBMSC-sheets showed strong green fluorescence *in vitro*. The green fluorescence of rADSC- and rBMSC-sheet and patches was observed *ex vivo* and an hour after and a day after stem cell-sheet transplantation.

Figure 4a shows the process of attaching cell sheets to the pancreas resection site. The pancreatic juice was secreted only where the duct was opened, which is the optimal location for attaching the cell sheet. After DP, the stem cell sheets were attached using CellShifter^TM^ on the resection surface. After five minutes, which is a sufficient time for the cell sheet to attach to the resection site, the shifter was removed, leaving behind only the cell sheet. Figure 3b shows the GFP fluorescence of the cell sheets. To confirm the continued attachment of rADSC and rBMSC sheets on the pancreatic resection site, GFP was imaged one hour and one day after cell sheet transplantation. Strong green fluorescence of the GFP cells was detected on the pancreas resection site at one hour and one day post-operation. No significant difference in GFP fluorescence was observed between the transplanted rADSC- and rBMSC-sheet patches.

### Efficacy of cell sheets for pancreatic leakage prevention

**Figure 5:**
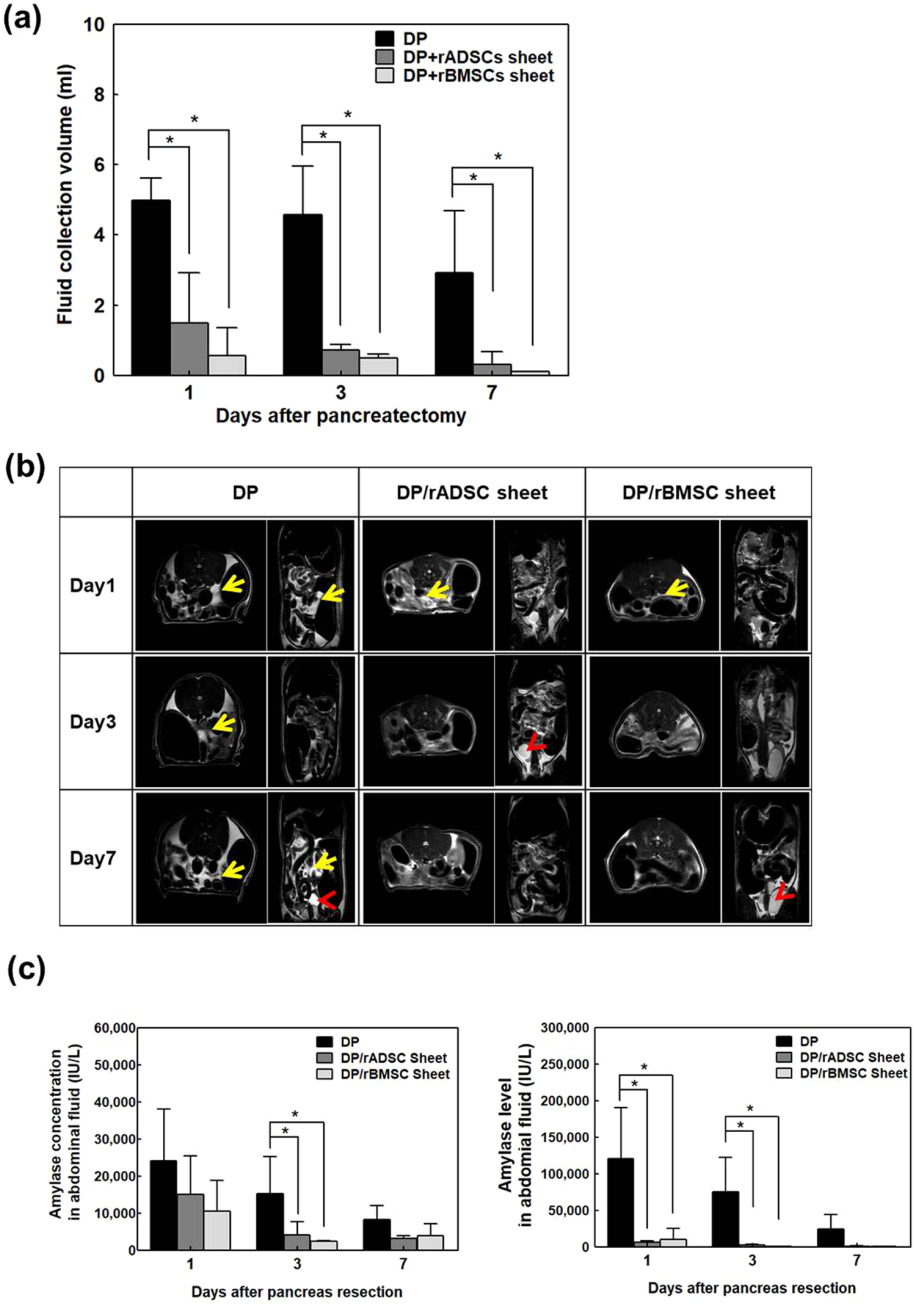
Efficacy of stem-cell sheets patches to prevent POPF. Control group DP model without stem cell sheet patches), DP with rADSC-sheet patches, and DP with rBMSC-sheet patches was compared experimentally. (**a**) The abdominal fluid was retrieved from each group on days 1, 3 and 7 after pancreatectomy (±SD n = 5, each). *P < 0.05. (**b**) Representative MR imaging of each group. The right panel of each image is the transverse section, and the left panel is the longitudinal section. The yellow arrow indicates the abdominal fluid (white area). Yellow arrowhead indicates water in the kidney. (**c**) Amylase concentration was analyzed on days 1, 3, and 7 to confirm the presence of pancreatic juice in the abdominal fluid. Amylase concentration decreased over time in all groups. Amylase concentration tended to increase in the control group compared to rADSC-sheet patch and rBMSC-sheet patch groups. Total amylase level was calculated by multiplying the amylase concentration and the abdominal fluid volume (±SD, n = 5, each).

The DP model without cell sheet attachment (control), the DP model with rADSC sheet patches, and the DP model with rBMSC sheet patches were compared experimentally. To confirm the efficacy of stem cell sheet patches in preventing pancreatic juice leakage, abdominal fluid volume was collected and measured on days 1, 3 and 7. As shown in Fig. 5a, abdominal fluid retrieved from the control group was 4.99 ± 0.63 ml on day 1, 4.58 ± 1.39 ml on day 3, and 2.93 ± 1.76 ml on day 7 (n = 9). Abdominal fluid retrieved from the rADSC-sheet patch group was 1.50 ± 1.43 ml on day 1, 0.71 ± 0.16 ml on day 3, 0.31 ± 0.31 ml on day 7 (n = 9), and from the rBMSC-sheet patch attachment group was 0.55 ± 0.80 ml on day 1, 0.50 ± 0.10 ml on day 3, and 0.10 ± 0.00 ml on day 7 (n = 9). There was a significant reduction in fluid collection from both rADSC- and rBMSC-cell-sheet patch groups compared to the control group (Control vs. rADSC, p < 0.001 on day 1 and day 3; p = 0.005 on day 7. Control vs rBMSC; p < 0.001 on day 1 and day 3; p = 0.002 on day 7). There was no significant difference between rADSC and rBMSC-sheet patch groups on day 1 and 7 (p = 0.101 at day 1 and p = 0.108 at day 7). However, the rBMSC-sheet patch group had a smaller ascites volume than the rADSC sheet-patch group on day 3 (p = 0.004).

MR imaging showed a similar tendency regarding the ascites volume level. MR scans were taken on day 1, 3, and 7 for the control group and the cell-sheet patch groups. The fluid collection area is shown in ImageT2 which appeared bright (Fig. 5b). For the longitudinal section, the fluid collection area was measured in the kidney image. For the transverse image, the liver was sectioned from the top at 3 mm intervals and a total of 30 images were obtained. The control POPF rat showed abdominal fluid on day 1, 3 and 7 days after surgery, as shown in the white area, while the rats with rADSC and rBMSC-sheet patches showed considerably less abdominal fluid after surgery.

### Measurement of amylase level from abdominal fluid collection

To confirm the presence of pancreatic juice in the abdominal fluid collection, the amylase concentration and level were measured (Fig. 5c). Amylase concentration decreased over time in all groups. Amylase concentration tended to increase in the control group compared to the rADSC-sheet patch and rBMSC-sheet patch groups. The amylase concentration of the control group was significantly higher than that of both stem cell-sheet patch groups on day 3 (p = 0.015 the rADSC-sheet patch group and p = 0.042 for the rBMSC-sheet patch group). According to the reference, mean drain amylase level is elevated more than 3 times than serum level compatible in the definition of a chemical pancreatic leak in ISGPF rather than just pancreatitis^16^. The normal value of amylase concentration in serum was 748 ± 71 IU/L in our experiment. Amylase concentration in the abdominal fluid in all groups were elevated more than 10 times than normal level at day 1. While amylase concentration in sheets groups decreased with time and reached about 5 times of normal level at day 3 and 7, amylase concentration in DP group was still more than 10 times higher than normal. No significant difference in the amylase concentration and level was observed between the rADSC and rBMSC sheet patch groups.

### Body weight and hematological changes after distal pancreatectomy

**Figure 6:**
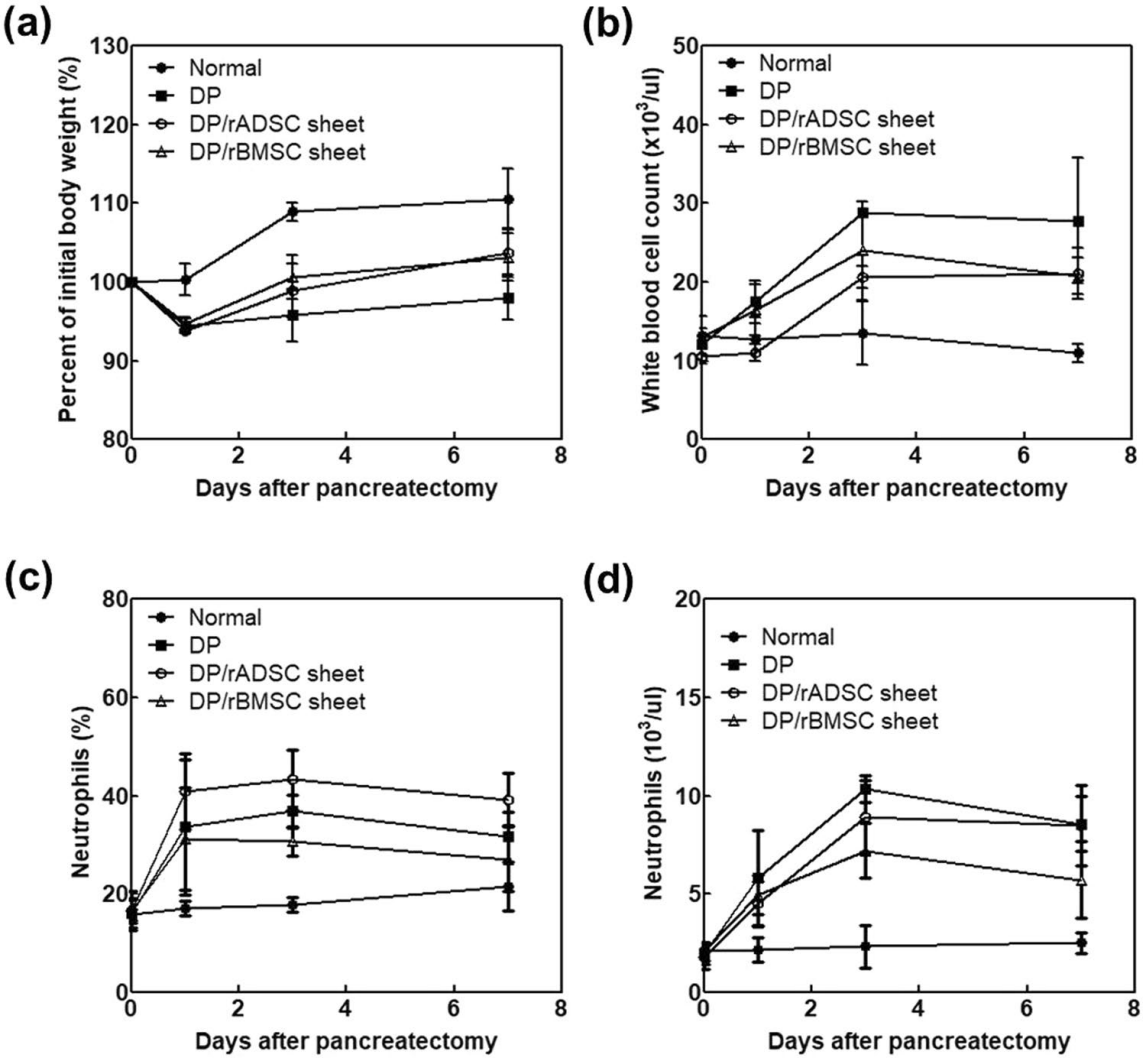
Body weight and hematological changes after distal pancreatectomy. Normal rats, control group (DP model without cell sheet attachment), DP with rADSCs sheet attachment, and DP with rBMSCs sheet attachment were compared. (**a**) Body weight changes after distal pancreatectomy on days 0, 1, 3 and 7 to confirm the feed intake or pancreatitis. (**b**) White blood cell (WBC) count, (**c**) Neutrophils (%) and (**d**) Neutrophils count in differential leucocyte count to confirm inflammation after pancreatectomy.

At day 1 after distal pancreatectomy, weight loss occurred in all experimental groups. In the case of the sheet groups, the body weight tended to gradually increase until day 3 and 7, but the reduced body weight in DP group was not recovered during the first week (Fig. 6a). Similarly, inflammation-related white blood cells (Fig. 6b) and neutrophils (Fig. 6c and d) increased in all animals immediately after surgery and then gradually decreased. The BMSC sheet group showed the fastest decline, while the DP group maintained high white blood cell and neutrophil counts, albeit without statistical significance.

### Immunomodulatory effects of rADSC-sheet and rBMSC-sheet patches on the DP

**Figure 7:**
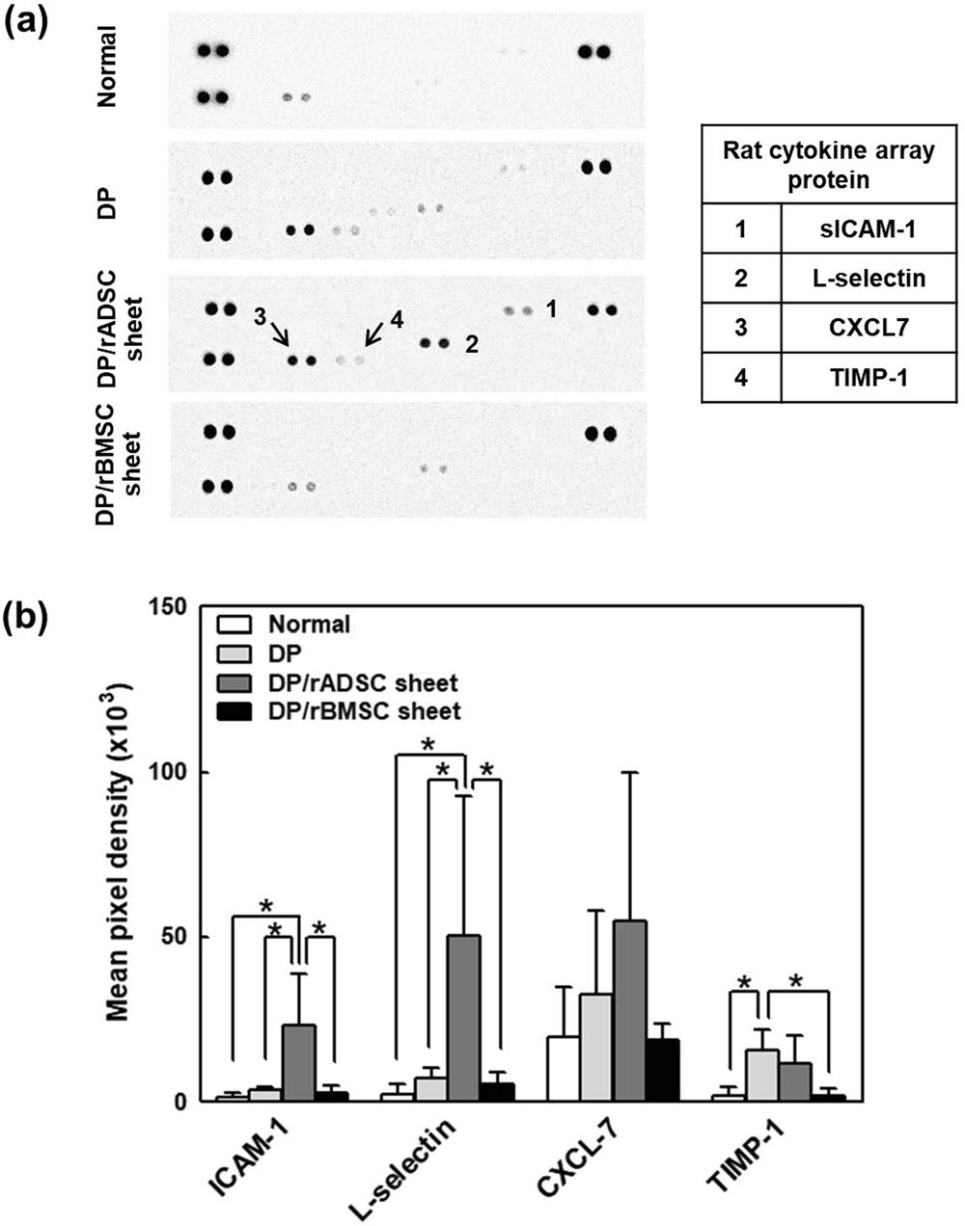
Rat cytokine array for detection of immunomodulatory effects of the rADSC-sheet and rBMSC-sheet patches on the pancreas resection site. Pancreas remnant of untreated rats (normal), DP control, rADSC-sheet patch attached with DP, and rBMSC-sheet patch attached with DP on day 7 post-operation. (**a**) Representative membranes and (**b**) selective quantification are shown for the tissues of each group. Selective quantification markers include soluble ICAM (CD54), LIX, L-selectin, thymus chemokine, and TIMP-1. All experiments were performed at least three times.

Immune responses of rADSC- and rBMSC-sheet patches were examined with an immune array kit in order to determine inflammation and pancreatitis. On day 7 post-DP, the area 3 mm toward the pancreatic head from the pancreatic resection surface was removed from normal, DP control, DP with rADSC-sheet patch group, and DP with rBMSC-sheet patch rats. In the DP group, adhesion and immune markers, including soluble ICAM (CD54), LIX, L-selectin, Thymus chemokine, TIMP-1, showed increased levels compared to normal rats. The rADSC-sheet patch group showed higher levels of soluble ICAM-1 (CD54), L-selectin, thymus chemokine, and TIMP-1 compared with the other groups. The BMSC-sheet patch group showed no expression or significantly lower levels of these proteins compared with the control group and the rADSC-sheet-patch group (Fig. 7).

### Immunohistochemistry and H&E staining of stem-cell-sheet patch groups

**Figure 8:**
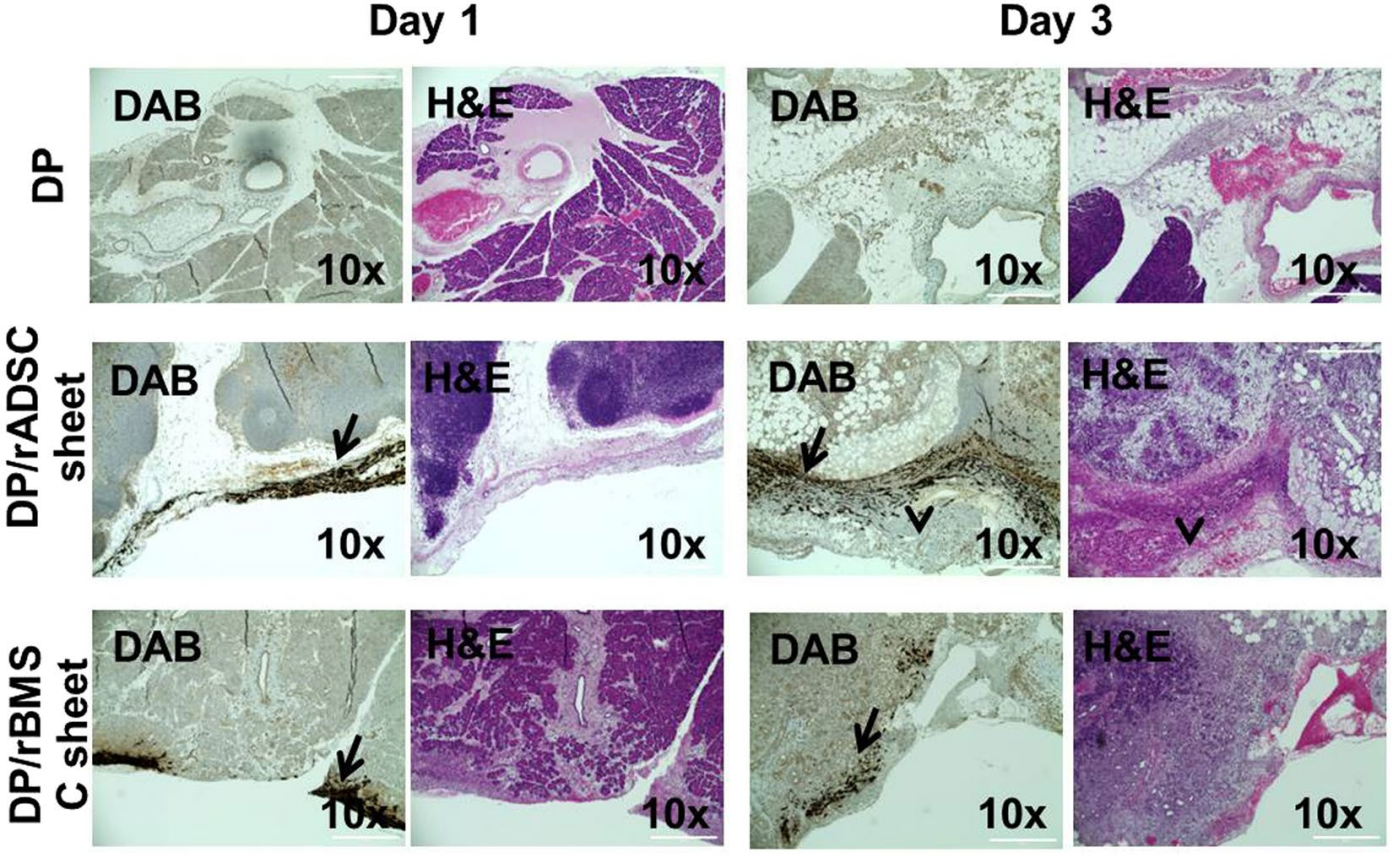
Histological and immunohistochemical analysis of the pancreas of the DP-only group and rADSC- and rBMSC-sheet patch groups after DP on days 1 and 3. (**a**) Immunohistochemical staining for GFP in the rADSC-sheet patch and rBMSC-sheet patch groups. Arrow indicates transplanted stem-cell sheets on the resection surface. Scale bars represent 400 μm. GFP cells that adhered to the ducts were observed in rADSC- and rBMSC-sheet patch groups on day 1 and 3. (**b**) H & E staining of IHC-stained tissues. While all groups showed inflammatory-cell accumulation at the resection site on day 1, the stem-cell sheet patch groups showed reduced inflammatory responses and regeneration started to occur around the patch site on day 3. A dense layer of regenerated tissue and adhesion to other organs to the exposed surface of the rADSC-sheet patch was observed (arrow ahead).

To confirm that the stem cell-sheet patches remain attached to the resection surface for prolonged periods, the transplanted cells were detected with GFP fluorescence. Many tissues and blood cells had autofluorescence; therefore, in order to remove autofluorescence and specifically identify the transplanted cells, GFP was identified by immunohistochemical (IHC) staining. When the tissues were examined on 1 and 3 days post-operation, adherence of the stem-cell sheet patches to the duct was observed (Fig. 8). All groups, including DP, rADSC-sheet patch with DP, and rBMSC-sheet patch with DP, showed inflammatory cell accumulation on the resection site. The presence of an extracellular matrix barrier on day 1 was confirmed. On day 3, the stem-cell-sheet groups had decreased inflammatory responses, and regeneration started to take place around the transplantation sites. A dense layer of regenerated tissue and adhesion of other organs to the exposed surface of the transplanted rADSC sheet patches were observed.

## Discussion

While there has been significant improvement in pancreas surgical techniques, pancreatic fistula still occurs at a high rate. POPF is a life-threatening complication that significantly prolongs hospital stay and increases healthcare costs^17^. Substantial effort has been made to prevent pancreatic fistula, such as modifying the surgical procedure and drainage regime, and selecting materials that can minimize the occurrence of POPF^3–5,18,19^; however, a definitive approach for preventing POPF is still lacking^6,7^. Non-resorbable adhesives such as neoprene or prolamine are commonly used in clinical practice to prevent the leakage of pancreatic juice; however, they are limited in that permanent closure can cause pancreatic atrophy and complete loss of exocrine function^20^. Fibrin glue sealant or other topical hemostatic agents have been used for pancreatic anastomosis, and polyglycolic acid felt may reduce pancreatic anastomotic leak. However, recent large-scale meta-analyses that assessed the effects of fibrin glue on pancreatic surgery reported that there were no significant effects^21–25^. Also, one report showed that fibrin glue followed by wrapping of the PGA mesh around the remnant pancreatic stump is associated with decrease in clinical POPF; however, other studies have reported that this procedure only reduces the severity, and not the incidence of POPF^26^. These controversial results indicate that the current materials in use are not sufficient for preventing pancreatic fistula. These materials are difficult to be applied onto wet and rough surfaces, and simply serve as a passive barrier for inflammation and fluid intake rather than inducing tissue regeneration. The characteristics of an ideal material for preventing POPF include flexibility, biocompatibility, adhesiveness, and ability to induce wound healing at the resection site. The physical and biological properties of materials should be carefully considered prior to clinical application. In this regard, the application of MSCs for preventing POPF is a promising approach due to their high regeneration ability and immune modulation function.

Recently, many clinical studies have reported the successful clinical application of MSCs for wound healing^27–29^ and immune diseases^30^. Despite the advantages of cell therapy using MSCs, traditional single cell injection method is difficult to be applied to local regions because of massive cell loss and low survival rate of cells. Hence, a novel technique for efficiently transplanting cells to target regions is needed. Cell sheet technology is advantageous since cell sheets are harvested without the use of damaging enzymes, which enables them to maintain their shape and characteristics. The sheet’s adhesion proteins facilitate attachment to the tissue surface, and the cell-cell interaction within the sheet makes them respond similarly to a tissue^11,12^.

For the present study, it was very important to develop a clinically-relevant pancreatic leakage animal model. In order to do so, we injured the splenic and gastric duct (model II) at a position close to the portal vein and performed DP and splenectomy. We then injured the splenic and gastric duct (model II) at a position close to the portal vein and performed DP and splenectomy. This approach is different from that of a recent study which involved cutting the splenic duct and leaving the pancreas itself intact^31^. Neither recent animal model can fully represent clinical POPF, but model II is the most accurate model available at this time.

POPF includes not only pancreatic leakage of postoperative pancreatic juice, but also various life-threatening clinical consequences such as intraperitoneal inflammation, abscess with or without sepsis, and delayed gastric emptying^32^. For clinical application, these clinical symptoms must be verified. In the present study, we focused on the reduction of pancreatic leakage and amylase level considering the small size of the experimental animals. In our experiment, there was a significant difference in the amount of fluid and amylase level between the control group and the sheet application groups. Both kinds of cell sheets demonstrated potential in preventing pancreatic leakage. Although not fully representative of the clinical situation, we have found that the weight reduction and inflammatory processes such as white blood cell and neutrophil counts were less in the sheet groups than in DP group. Thus, the sheet groups showed less feeding problems and inflammatory reactions.

In the present study, we isolated MSCs from two different tissues; adipose tissue and bone marrow. Adipose derives stem cells, which are often used in regenerative therapies, can repeatedly be acquired and grow rapidly^33^. They are multipotent cells and secrete a wide range of regenerative factors. Bone marrow-derived stem cells are another viable source of mesenchymal stem cells for cellular therapy. They are reported to have exceptional potential in differentiation and immune modulation abilities^34–36^.

To monitor the attachment and survival of the stem cell-sheet, we isolated stem cells from GFP transgenic rats. The transplanted rADSC- and rBMSC-sheet patches remained attached to the resection site. After three days, rADSC and rBMSC were not only attached, but were also proliferating on the surface. The rACSC-sheet patches had a high degree of adherence to surrounding organs and tissues in the abdomen^37^. We suspect that these results are relevant to the studies that suggest ADSC recruit adhesion and regeneration factors such as FGF-2, VEGF, and HGF^38^. In contrast, rBMSC-sheet patches were not adhesive to other organs. This difference in adhesion of the two types of patches to other organs will be investigated in future studies. A similar tendency was demonstrated in the immune cytokine array as well.

The results of cytokine and chemokine array indicated an increase in soluble ICAM-1, a type of adhesion protein, in the rADSC-sheet patch group only. Also, the rADSC patch group showed an increase in the levels of other molecules related to wound healing and immune cytokines such as L-selectin, CXCL7, and TIMP-1. rBMSC sheet patches had fewer immune reactions compared to control and rADSC sheet patch group. MSCs actively respond to stress, apoptosis, and inflammatory response in damaged tissues; MSCs are also known to promote angiogenesis, regeneration, immune cell activation or inhibition, and cell recruitment^36,39–41^. MSC-based tissue regeneration involves regulation of extracellular matrix precipitation, collagen synthesis, fibroblast proliferation, platelet activation, fibrinolysis, and angiogenesis^42^. The immune process often involves T cell inhibition, macrophage activation, and potential neutrophil replenishment. The results of the present study show that MSC-sheet patches are a promising approach for preventing pancreatic leakage. Also, MSC induces regeneration and immune modulation at the resection site. In this study, ADSCs were more likely to induce wound healing, and BMSCs tended to decrease inflammatory response.

The results of the present study show that MSC-sheet patches are a promising approach to prevent pancreatic leakage. In addition, MSC induce regeneration and immune modulation at the resection site. However, as reported in previous studies, the characteristics of MSCs vary greatly depending on culture conditions, tissue source and donor characteristics, and inflammatory status of the graft site. More extensive research is needed to reveal the mechanism of MSC sheet action *in vitro* and *in vivo*, and long-term follow-up of the transplanted cells is necessary. We also plan to test our experimental design in a porcine model to further determine its clinical potential.

## Materials and Methods

### Fabrication of mesenchymal stem cell sheets

rADSC and rBMSC stem cells were isolated from transgenic green-fluorescent-protein (GFP)-expressing seven-week SD-type rats, SD-TG (CAG-EGFP) (Japan SLC, Hamamatsu, Japan). Adipose tissue was harvested from the inguinal region and digested in 0.075% collagenase type I solution (Worthington, NJ, USA). rBMSC were isolated by collecting attached cells after flushing the marrows of the femur and tibia. rADSC and rBMSC were cultured in Dulbecco’s Modification of Eagle’s Medium (DMEM) mixed with 10% fetal bovine serum and 1% anti-antibiotics (GIBCO, MD, USA) in a 37 °C, 5% CO_2_ chamber.

The rADSC and rBMSC were cultured on temperature-responsive dishes (3.5 mm UpCell:^™^ Thermo Fisher Scientific, MA, USA) to form rADSC and rBMSC sheets, respectively. rADSC or rBMSC (1.1 × 10^6^) at passage 3 were seeded and cultured for two days. The sheets were transferred to a lower temperature chamber at 20 °C an hour before being transplanted to the rat pancreas. Figure 1a shows the scheme of rADSC and rBMSC isolation and formation of cell sheets (rADSC sheet: left panel and rBMSC sheet: right panel). The UpCell dishes are hydrophobic at 37 °C, but become hydrophilic at 20 °C which allows the cell sheets to detach from the dishes, while maintaining their cell-cell junctions and adhesion molecules (Fig. 1b). The cell sheets were attached to CellShifter^™^ membranes (Thermo Fisher Scientific, MA, USA) that were then transplanted onto the pancreas-resection site. After five minutes, which is a sufficient time for the cell sheet to attach to the resection site, the shifter was removed, leaving behind only the cell sheet (Fig. 1c).

### Establishment of rat model of postoperative pancreatic fistula (POPF)

SD-Rats (ORIENT BIO, Seongnam, South Korea) between 8–12 weeks old were used for the POPF experiments. This study was reviewed and approved by the Institutional Animal Care and Use Committee (IACUC, No. 2016–02–125) Asan Institute for Life Sciences, Asan Medical Center. The committee abides by the Institute of Laboratory Animal Resources (ILAR) guide. All experiments related to animals were performed in accordance with the relevant guidelines and regulations. Three different rat POPF models were developed: (1) model I (common pancreatic duct division): division of the pancreas where the common duct of the pancreas and bile duct intersect; (2) model II (gastric and splenic-duct division with distal pancreatectomy and splenectomy): division of both gastric duct and splenic duct at the left margin of the portal vein; and (3) model III (splenic duct division with distal pancreatectomy and splenectomy): removal of splenic duct at the pancreas tail level from spleen. The survival rates and abdominal fluid volumes of these three models were compared for 10 days after pancreas resection.

The experimental procedure was as follows: Rats were anesthetized and placed in the supine position. A midline incision was performed on the abdomen. By holding the stomach with an atraumatic forceps, the duodenum and spleen were exteriorized. Through omentectomy, the stomach and spleen were mobilized and the short gastric vessel was separated after ligating with a black silk 4-0 tie. The portal vein was exposed once the area between the colon and pancreas was mobilized. After determining the resection surface of the pancreas left of the portal vein, the resection surface was fixed with forceps, and the pancreas parenchyma was held with different forceps. The vessels were then tied with black silk 7-0 and resected. The ducts were left divided. The survival rate for up to 10 days for all three models and the volume of postoperative fluid were measured (n = 10).

### Characterization of mesenchymal stem cells sheet

To determine the MSC characteristics of the rADSC and rBMSC at passage 3, we examined the expression of MSC surface markers. After cells were blocked with 2% BSA, cells were incubated on ice for 1 hr at 4 °C with the following anti-human antibodies; PE-anti-rat CD29, PE-anti-rat CD 31, PE anti-rat CD45 (BD PHARMINGEN, NJ, USA); IgG FITC-isotype control, FITC-anti-rat MHC CLASS I, FITC-anti-rat CD90 (Abcam, Cambridge, UK) by 1:1000. The antibodies were incubated at 4 °C for 30 minutes and washed with PBS. Cells were analyzed by flow cytometry (FACS Caliber, Canto: BD Bioscience, San Jose, CA, USA).

To induce osteogenic and adipogenic differentiation, cells were plated at 4,000 cells/cm^2^ with culture medium and were cultured until confluency. At this point, the culture medium was replaced with either the adipogenic or osteogenic differentiation media (hMSC Osteogenic Differentiation BulletKit Medium or hMSC Adipogenic Differentiation BulletKit Medium; Lonza Japan, Tokyo). After 14 days in culture, the adipogenic culture formed adipose-like vacuoles. The plates were fixed and stained with Oil Red O (Sigma-Aldrich, St Louis, MO, USA). The osteogenic differentiation cultures were incubated for 28 days; the cells were then fixed and stained with 1% Alizarin Red solution pH 4.1 (Sigma-Aldrich, St Louis, MO, USA). The green fluorescence of the stem cells from SD-TG rats (CAG-EGFP) was confirmed by fluorescence microscopy (EVOS^TM^ FL Auto Imaging System, ThermoFisher Scientific, MA, USA). To stain fibronectin of the cell sheet, the cell sheet was embedded in paraffin and sliced into 4 um-thick cross-sections. The sections were stained with primary fibronectin antibody (Santa Cruse Biotechnology, Inc., CA, USA) and secondary anti-mouse Goat Anti-Mouse Alexa Fluor® 555 (Thermo Fisher Scientific, MA, USA) at 1:100 dilution.

### Optical imaging of the GFP rADSC and rBMSC sheet

To observe the attached GFP-expressing rADSC- and rBMSC-sheet patches on the pancreatic resection site, GFP expression of rADSC and rBMSC was detected with an IVIS Spectrum system (Caliper Inc., Alameda, CA). For organ (*ex vivo*) imaging, fresh organs were placed on plates and analyzed. EGFP was excited at 488 nm (filter range 445 to 490 nm) and detected at 510 nm. The region of the interested (ROI) level was measured with radiance (photons/s/cm^2^/sr) using an analysis program, Living Image 4.4 (Caliper Life Sciences, PerkinElmer Inc.).

### Magnetic resonance (MR) imaging

9.4 T/160 mm scanner (Agilent Technologies, Palo Alto, CA, USA) with 400 mT/m gradient sets was used for MR imaging of fluid collection in the abdomen. Transverse images from the top of the liver to the bottom of the bladder were sliced into 30 pages of 1.5 mm thickness (matrix = 192 × 256; field of view = 3.0 × 4.0 cm; section thickness = 1.5 mm; section gap = 0.33 mm; and a number of sections = 30 sections with the longitudinal axis on the bottom). Longitudinal images from the top of the abdomen to the bottom were sliced into 20 pages of 3.0 mm thickness (Sequence parameters: matrix size = 192 × 256; field of view = 4.5 × 5.5 cm; section thickness = 3.0 mm; section gap = 0.5 mm). Locations of the organs and fat were obtained in the T1 image, and images of water were obtained in T2. T2 images were analyzed with the program image J (DICOM image by National Institutes of Health, MD, USA)^43,44^.

### Amylase assay in abdominal fluid

Following euthanasia, rat abdominal fluid was collected via a 10 ml syringe after sacrifice and transferred into a tube. Amylase concentration was measured with an amylase activity kit (Abcam, Cambridge, UK). The assay procedures were followed from the manufacturer’s instructions. Amylase level was calculated by multiplying the amylase concentration by the abdominal fluid volume.

### Leukocytes count

Blood samples were collected for hematology determinations in tubes with anticoagulants (EDTA-2 K). Hematology determinations included white-blood-cell (WBC) count and differential leucocyte count (neutrophils, lymphocytes, monocytes) using an Advia 120 Hematology Analyzer (Bayer Healthcare, Myerstown, PA, USA).

### Cytokine array of tissues

Rat cytokines and chemokines array kit (R&D SYSTEMS, MN, USA; #ARY008) was used to carry out immune cytokine analysis. The tissue section 3 mm towards the pancreas head from the pancreas resection surface was used. The tissue was soaked in PBS with protease inhibitor (SIGMA-Aldrich) and homogenized. The supernatant was used for analysis. 400 µg of protein was used per membrane based on protein quantification. The subsequent steps were directed by the manufacturer’s instructions. The experimental groups were as follows: normal rats that did not undergo any operation; a group that only underwent distal pancreatectomy (DP); and two experimental groups in which rADSCs sheets and rBMSCs sheets were attached to the resection surface after DP, respectively. Interleukins, activators of B lymphocytes, activations of natural killers, and multiple biological effectors were identified with this kit.

### Immunohistochemistry and H&E staining

The rats were sacrificed either on day 1 or 3, and each pancreas was removed. Formalin-fixed, paraffin-embedded sections (4 μm in thickness) were deparaffinized, dehydrated through a graded alcohol series. To perform hematoxylin and eosin (H&E) staining, samples were deparaffinized and dehydrated, followed by applying hematoxylin (Sigma-Aldrich, MO, USA) and eosin staining (Sigma-Aldrich, MO, USA). Immunohistochemistry was performed using primary antibodies for GFP (dilution 1:1000, Abcam, Cambridge, UK). Dehydrated samples were blocked with hydrogen peroxide, and dried for 10 minutes at RT then for 20 minutes in an incubator at 65 °C. An automated slide preparation system (Benchmark XT; Ventana Medical Systems Inc., Tucson, AZ, USA) with OptiView DAB Detection Kit (Ventana Medical Systems) was used for immunohistochemistry.

### Statistical analysis

Statistical significance was determined using GraphPad Prism 5 (GraphPad Software, San Diego, CA, USA). Statistical significance of the differences between groups was analyzed with the Student’s *t*-test and a two-way analysis of variance. P < 0.05 was used as the cut-off for determining statistical significance. The data are presented as the mean ± standard deviation, with the number of samples indicated in the figure legends.

## Electronic supplementary material

Supplementary figure 1
