# Supplementary material for: Engineered mesenchymal stem-cell-sheets patches prevents postoperative pancreatic leakage in a rat model
Source: Sci Rep. 2018 Jan 10;8:360. doi: 10.1038/s41598-017-18490-9 (PMC5762914; doi:10.1038/s41598-017-18490-9)

Supplementary Information for

**Engineered mesenchymal stem-cell-sheets patches prevents postoperative pancreatic leakage in a rat model**

Seong-Ryong Kim^1,+^, Hye-Jin Yi^2,+^, Yu Na Lee^2^, Ji Yoon Park^2,3^, Robert M. Hoffman^4,5^,

Teruo Okano^6^, In Kyong Shim^2,*^ & Song Cheol Kim^2,7,*^

^1^Department of Surgery, Division of HBP and Liver Transplantation, Korea University Anam Hospital, Seoul, Korea

^2^Asan Institute for Life Sciences, Asan Medical Center, University of Ulsan College of Medicine, Seoul, Korea

^3^Department of Chemistry, Wesleyan University, Connecticut, United States

^4^Department of Surgery, University of California, San Diego, CA, USA

^5^AntiCancer Inc., San Diego, CA, USA

^6^Institute of Advanced Biomedical Engineering and Science, Tokyo Women's Medical University, Tokyo, Japan

^7^Department of Surgery, Asan Medical Center, University of Ulsan College of Medicine, Seoul, Korea

Figure S1. Full-length blots displayed in Fig. 6a


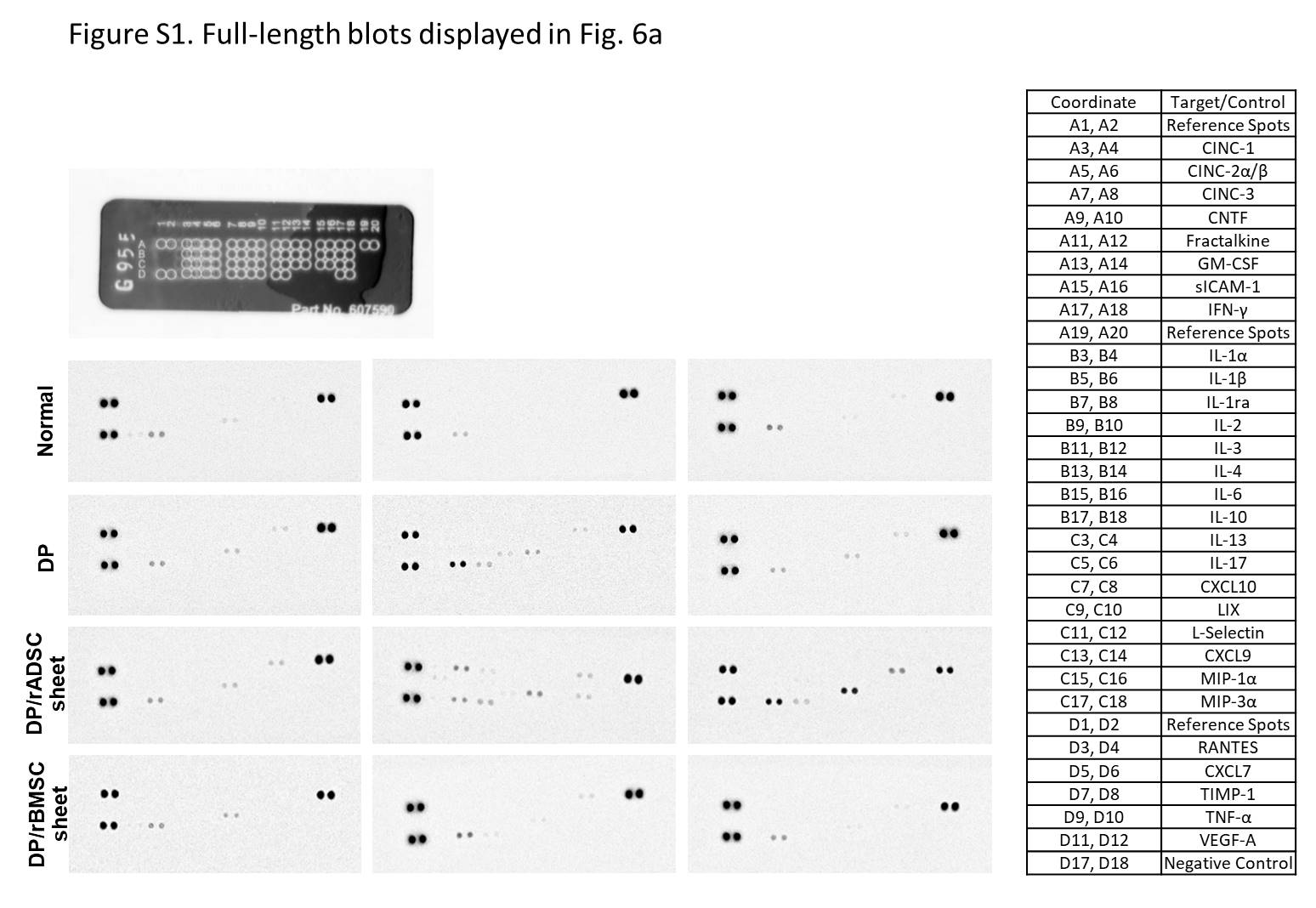

Supplement: Supplementary file 1 — Supplementary figure 1 [file 41598_2017_18490_MOESM1_ESM.doc]
